# Starch Flocculation by the Sweet Potato Sour Liquid Is Mediated by the Adhesion of Lactic Acid Bacteria to Starch

**DOI:** 10.3389/fmicb.2017.01412

**Published:** 2017-07-25

**Authors:** Lili Zhang, Yang Yu, Xinhua Li, Xiaona Li, Huajiang Zhang, Zhen Zhang, Yunhe Xu

**Affiliations:** ^1^Department of Food Science and Engineering, Jinzhou Medical University Jinzhou, China; ^2^Liaoning Provincial Research Center of Meat Processing and Quality Control Jinzhou, China; ^3^Department of Food Science, Shenyang Agricultural University Shenyang, China; ^4^Department of Food Science, Northeast Agricultural University Harbin, China

**Keywords:** lactic acid bacteria, starch flocculation, starch-binding proteins, sweet potato sour liquid, natural fermentation

## Abstract

In the current study, we focused on the mechanism underlying starch flocculation by the sweet potato sour liquid. The traditional microbial techniques and 16S rDNA sequencing revealed that *Lactobacillus* was dominant flocculating microorganism in sour liquid. In total, 86 bacteria, 20 yeasts, and 10 molds were isolated from the sour liquid and only eight *Lactobacillus* species exhibited flocculating activity. *Lactobacillus paracasei* subsp. *paracasei* L1 strain with a high flocculating activity was isolated and identified, and the mechanism of starch flocculation was examined. *L. paracasei* subsp. *paracasei* L1 cells formed chain-like structures on starch granules. Consequently, these cells connected the starch granules to one another, leading to formation of large flocs. The results of various treatments of L1 cells indicated that bacterial surface proteins play a role in flocculation and L1 cells adhered to the surface of starch granules via specific surface proteins. These surface starch-binding proteins were extracted using the guanidine hydrochloride method; 10 proteins were identified by mass spectrometry: three of these proteins were glycolytic enzymes; two were identified as the translation elongation factor Tu; one was a cell wall hydrolase; one was a surface antigen; one was lyzozyme M1; one was a glycoside hydrolase; and one was an uncharacterized proteins. This study will paves the way for future industrial application of the L1 isolate in starch processing and food manufacturing.

## Introduction

The processing of starch flocculated with sour liquid has a 400-year history in China (Research Groups of Sour Liquid, 1974). Sour liquid is milky-white or yellowish-white, with a sour taste, and it is generated by natural fermentation. It is used as a flocculant, as it accelerates starch flocculation and shortens the settling time of starch (Research Groups of Sour Liquid, 1974; Wei and Qun, 2007). The technology of sour liquid-aided flocculation is mainly used for the processing of sweet potato starch or mung bean starch.

It has been reported that microorganisms present in the sour liquid are responsible for the flocculation of starch. *Streptococcus lactis* from a mung bean sour liquid was isolated, and shown to bind to the starch granules, and promote the flocculation of starch (Xu and Liu, 1980). In addition, flocculation is affected by temperature, pH, free ion concentration, and other factors (Research Groups of Sour Liquid, 1974). However, the mechanism by which *S. lactis* flocculates starch is still unclear. This restricts the application of the sour liquid technology in the processing of other plant starches or in large-scale industrialization.

Although the mechanism by which lactic acid bacteria bind to and flocculate starch remains unclear, the starch-binding activity of bacteria has been investigated in *Bifidobacterium, Bacteroides thetaiotaomicron, Lactobacillus amylovorus*, and *Vibrio cholerae* cells (Reeves et al., 1996, 1997; Crittenden et al., 2001; Rodriguez-Sanoja et al., 2005; Ryan et al., 2006; Niderman-Meyer et al., 2010). Researchers found that bacteria from the *Bifidobacterium* genus possess a strong starch-binding ability, and are absorbed and embedded in resistant starch granules (Crittenden et al., 2001). These characteristics have been exploited during preparation of probiotic microcapsules, markedly decreasing the difficulties associated with the production of these microcapsules (Crittenden et al., 2001). The starch-binding activity of *B. thetaiotaomicron* plays an important role in starch metabolism in the mammalian gut (Shipman et al., 2000; Crittenden et al., 2001). Drugs with resistant starch as an adjuvant are characterized by a relatively good efficacy in treating acute gastroenteritis caused by *V. cholerae*; this species can specifically bind to the surface of resistant starch granules, thus accelerating the discharge of *V. cholerae* from the body (Niderman-Meyer et al., 2010). The starch-binding activity of *B. thetaiotaomicron*, a Gram-negative bacterial species, is mediated by the outer membrane proteins SusC, SusD, SusE, and SusF (Donaldson et al., 2016; O’Toole, 2016). Similarly, starch binding by *Bifidobacterium* involves specific cell surface proteins rather than non-specific hydrophobic and electrostatic interactions; however, the property of proteins that participate in the adhesion of *Bifidobacterium* to starch remains unclear (Crittenden et al., 2001). Starch-binding activities of these bacteria are closely associated with their cell wall proteins (Shipman et al., 2000; Crittenden et al., 2001).

In the current study, we focused on the naturally fermented sour liquid of the sweet potato. The V4 regions of 16S rRNA genes of bacteria present in that liquid were analyzed by high-throughput sequencing, in conjunction with traditional microbial isolation and culture techniques, to determine the dominant microorganisms with starch-binding and flocculating activities. The mechanism of starch flocculation was then elucidated at a cellular level. Proteins that mediated the lactic acid bacteria binding to starch were identified by mass spectrometry. The results will provide theoretical basis for enhanced sour liquid application in the processing of starch for bean vermicelli production, and for the use of starch-binding lactic acid bacteria in food manufacturing.

## Materials and Methods

### Materials

The sweet potato sour liquid was obtained from Yingnahe Starchworks (Dalian City, Liaoning Province, China). The sour liquid (1 L) was collected in a sterile culture flask and transferred to a laboratory at 4°C. Microorganisms were plated for enumeration and isolation on the same day. Sweet potato starch was purchased from Shandong Bio Sunkeen, Co., Ltd. (Jining City, China). Phosphate-buffered saline (PBS, pH 7.2) was purchased from Sigma Chemical, Co. (St. Louis, MO, United States). All other chemical reagents were of analytical grade.

The sweet potato juice medium was prepared as follows. Sweet potato infusion was prepared by boiling 200 g of sliced (washed but unpeeled) sweet potatoes in 1 L of distilled water for 30 min, and decanting or straining the broth through cheesecloth. Distilled water was added such that the total volume of the suspension was 1 L; 20 g of glucose, 2 g of lactose, 5 g of yeast extract, and 5 g of sodium acetate was then added, and the medium was sterilized by autoclaving at 115°C for 15 min.

### Microorganism Counts and Isolation

Microorganisms were enumerated and isolated by serial dilution and plating. Bacterial counts and isolation were conducted on Tomato Juice Agar (TJA) media supplemented with cycloheximide (50 μg/mL) to inhibit fungal growth (Muyanja et al., 2003; Lin et al., 2006). The plates were incubated at 30°C for 24 h. Yeasts and molds were inoculated on Rose Bengal agar plates and incubated at 30°C for 3–5 days (Coombs and Franco, 2003). To distinguish between the two, colonies that were smooth and wet were considered as yeasts; downy or furry colonies were considered to be molds. Bacterial colonies were counted using automatic colony counters (Interscience Scan 1200). Colonies with distinct morphological characteristics were selected and transferred onto sweet potato juice slant medium (*vide infra*), cultured at 30°C (bacteria for 1 day, yeasts for 3 days, and molds for 5 days), and were then stored at -4°C to screen the strains with high flocculating activities (Anastasi et al., 2005).

### Screening Methods

The selected strain slopes were inoculated into 5 mL of sweet potato juice medium and cultured at 30°C. Bacteria and yeasts were cultured for 1 and 3 days, respectively. The molds were cultured with shaking at 160 rpm for 5 days. Then, the flocculation rate of fermentation liquor was determined as the flowing methods. Using the flocculation rate of the cultures as an index, with the sweet potato juice medium as a control, the strains were screened for high flocculating starch activity.

### Flocculation Rate (FR) Measurements

Distilled water (100 mL), 0.5 g of sweet potato starch, and 5 mL of the liquor to be tested were placed in a 150-mL beaker. The liquid was agitated for 3 min on a magnetic stirring apparatus, and then left to stand for 3 min. As a control, sweet potato juice was used instead of the fermented liquor. The flocculation efficiency was expressed as FR, by measuring the decrease of turbidity of the upper phase (Lian et al., 2008; Beck et al., 2009; Bhattacharya et al., 2017). FR was calculated by the following equation:

$$\begin{array}{c} \mathrm{FR}(\%)=\frac{A-B}{A}\times 100\% \end{array}$$

Where A and B are optical densities of the control and sample, respectively, at 550 nm.

### Bacterial Sampling for 16S rDNA Sequencing

Microbial genomic DNA was extracted from 1 mL of the sour liquid by using the TIANGEN DNA stool mini kit (TIANGEN, cat#DP328) according to the manufacturer’s instructions. The V4 variable region of 16S rDNA was amplified using the universal primers 520F (5′-AYTGGGYDTAAAGNG-3′) and 802R (5′-TACNVGGGTATCTAATCC-3′) (Blanton et al., 2016). The PCR amplification and the construction of a sequencing library were performed, as described previously (Xu et al., 2016). For each sample, barcoded V4 PCR amplicons were sequenced using the Illumina MiSeq platform (Dong et al., 2015; Donaldson et al., 2016). Amplification and sequencing of the V4 variable region of 16S rDNA was completed by Personal Biotechnology, Co., Ltd. (Shanghai, China).

Sequence reads were excluded from analysis if their length was less than 150 bp, the average Phred score was lower than 20, contained ambiguous bases, a homopolymer run exceeding six bases, or when mismatches in primers were detected. Sequences that passed quality filtering were then assembled by Flash^1^, which required that the overlap of reads 1 and 2 was ≥10 bp, without any mismatches. The reads that could not be assembled were discarded. Chimera sequences were removed using UCHIME in mothur (version 1.31.2^2^).

### Operational Taxonomic Unit (OTU) Clustering

Sequence clustering was performed using UCLUST algorithm in QIIME^3^; the sequences were clustered into operational taxonomic units (OTUs). The longest sequence in each cluster was selected as the representative. The taxonomy of each OTU was assigned by BLAST-searching the representative sequence against Greengenes reference database (Release 13.8^4^) (Xu et al., 2016).

### Strain Identification by 16S rDNA Sequencing

Pure isolates were grown to a late stationary phase in 5 mL of media. The cultures were centrifuged for 10 min at 4,000 × *g*. Each cell pellet was resuspended in 0.5 mL of dH_2_O, and DNA was extracted using the TIANGEN DNA stool mini kit (TIANGEN, cat#DP328) according to the producer’s instructions.

Full-length 16S rDNA amplicons were generated with bacterial primers 27F (5′-AGAGTTTGATCCTGGCTCAG-3′) and 1492R (5′-CTACGGCTACCTTGTTACGA-3′). The PCR amplification and sequencing were performed, as described previously (Piotrowska et al., 2016).

To identify 16S rDNA sequences most similar to the obtained sequences, all sequences were matched against nucleotide sequences deposited in GenBank using the BLASTn program^5^.

Finally, strain identification based on its colony character, morphological, and physiological characteristics, as well as 16S rDNA sequence homology referencing Bergey’s Manual of Systematic Bacteriology.

### Preparation of the *L. paracasei* subsp. *paracasei* L1 Fermentation Liquor

*Lactobacillus paracasei* subsp. *paracasei* L1 slope were inoculated into a tube of fermented sweet potato juice (5 mL) and were cultured for 24 h at 30°C. Then, the inoculum was inoculated into the sweet potato juice medium (5%, v/v) and cultured for 24 h at 30°C.

Flocculation activity of L1 in fermentation liquor was as follows. Five milliliter of the fermented liquor were centrifuged at 4,000 × *g* for 10 min. The cell pellet was washed twice with distilled water; 5 mL of distilled water was added to obtain a bacterial suspension. Distilled water (100 mL), 0.5 g of sweet potato starch, and 5 mL of the bacterial suspension were placed in a 150-mL beaker to test the flocculation activity.

### Flocculating Activity of *Lactobacillus paracasei* subsp. *paracasei* L1 in Fermentation Liquor

*Lactobacillus paracasei* subsp. *paracasei* L1 culture (10 mL) was centrifuged at 4,000 × *g* for 10 min. The cell pellet was washed twice with dH_2_O, and 10 mL of distilled water was added to obtain a bacterial suspension. Flocculating activities of culture broth, cell-free supernatant and cell pellet were tested (Lian et al., 2008).

### Determination of the Particle Size of Starch Granules before and after Flocculation

*Lactobacillus paracasei* subsp. *paracasei* L1 cultures (10 mL) were centrifuged at 4,000 × *g* for 10 min. The cell pellet was washed twice with distilled water; 10 mL of distilled water was added to obtain a bacterial suspension. Distilled water (100 mL), 0.5 g of sweet potato starch, and 5 mL of the bacterial suspension were placed in a 150-mL beaker. Microtrac laser particle size analyzer (S3500, American Microtrac Company) and laser diffraction particle size distribution meter were used to determine the particle size distribution of sweet potato starch before flocculation. Thereafter, the liquid was agitated for 3 min on a magnetic stirring apparatus, and then left to stand for 3 min. Microtrac S3500 was next used to determine the particle size distribution of starch and in the supernatant after flocculation (Biggs et al., 2000; Hjorth and Jørgensen, 2012).

### Microscopic Observation of Starch Granules with Adhered Bacteria

Sweet potato starch granules were observed by optical microscopy before and after the addition of *L. paracasei* subsp. *paracasei* L1 fermentation liquor. Samples of starch granules with adhered bacteria were fixed with a glutaraldehyde solution [3% (v/v) in 0.01 M phosphate buffer, pH 7.2] on brass stubs and chromium-coated by Xenosput 2000 chromium coater with the deposition parameters of 0.06 sputter Amps for 40 s. Coated preparations were visualized with Hitachi S4800 scanning electron microscope (SEM; Japanese Hitachi Ltd) at the accelerating voltage of 2 kV (O’Riordan et al., 2001).

### Determination of Zeta (ζ) Potential during the Flocculation Process

Sweet potato starch milk (100 mL) was weighed and tested by zeta potentiometer (nano-ZS, British Malvern). The values of ζ potential of sweet potato starch milk, *L. paracasei* subsp. *paracasei* L1 suspension, and sweet potato starch milk supplemented with 10% of *L. paracasei* subsp. *paracasei* L1 cells were determined (Hjorth and Jørgensen, 2012).

### Determining the Effect of Physical, Chemical, and Enzymatic Treatments on the Flocculating Activity of *L. paracasei* subsp. *paracasei* L1 Cells

*Lactobacillus paracasei* subsp. *paracasei* L1 cells were cultured for 24 h in the sweet potato juice medium, washed twice with PBS, and collected by centrifugation. The specific experiments were performed as follows: cells were resuspended in PBS to 8 log CFU/mL and then heat-treated in a water bath at 30, 40, 50, and 60°C for 30 min. Next, cells were resuspended in PBS to 8 log CFU/mL and then irradiated using an ultraviolet lamp (18 W, 15 cm, 3 h). Cells were resuspended in PBS to 8 log CFU/mL and then placed in an ice-water bath and sonicated for 5 min (CFS-25A-ultrasonic generator 8.6 kc, 250 W). Processing was stopped when the temperature reached values ≥ 15°C. When cells cooled to below 10°C, they were again treated for 5 min. When cells subsequently reached a temperature of 10°C, treatment was repeated for 5 min. Cells were pretreated with 3% trichloroacetic acid or 10^-4^ mol/L lithium chloride at 28°C for 30 min. Cells were resuspended in PBS to 8 log CFU/mL and then separately pretreated with the following enzymes: trypsin (from bovine pancreas, Sigma; 3 mg) ml^-1^, at pH 7.5 for 6 h at 37°C; α-amylase (from *Bacillus licheniformis*; 2 mg) ml^-1^ at pH 7.0 for 4 h at 40°C; lysozyme (from egg white; 1 mg) ml^-1^ at pH 6.0 for 1 h at 37°C. The flocculating experiment was performed using 3 g of Tween 80 L^-1^. Then, the flocculating experiment was performed using 5 g/L of glucose or maltose (O’Riordan et al., 2001; Wei and Qun, 2007).

### Isolation of Starch-Binding Proteins

*Lactobacillus paracasei* subsp. *paracasei* L1 was grown overnight in sweet potato juice medium, centrifuged (10,000 × *g*, 15 min, 4°C), and washed three times with PBS. The cells (1 g) were incubated in 20 mL of 4 M guanidine hydrochloride with shaking (at 200 rpm) for 60 min at 37°C. The supernatant was collected after centrifugation at 12,000 × *g* and 4°C for 10 min; it was dialyzed overnight in a dialysis bag, with PBS as the dialysis solution. PBS was replaced 5–6 times. Sweet potato starch (0.5 g) were added to 10-mL samples of the supernatant, shaken for 30 min to ensure full exposure of granule surface to the supernatant, and then washed with PBS and centrifuged (10,000 × *g*, 5 min, 4°C), three times, to remove unbound proteins. To extract starch-bound proteins, each starch pellet was resuspended and incubated for 5 min in PBS (control) and PBS containing 100 mM maltose. The starch was removed by centrifugation and the supernatant from each tube was separated and purified by sodium dodecyl sulfate polyacrylamide gel electrophoresis. The purified samples were digested with trypsin, analyzed by high performance liquid chromatography-electrospray ionization tandem mass spectrometry (LC-ESI-MS/MS) using Q Exactive (Thermo Scientific), and identified by Mascot 2.3.0 using the Uniprot *Lactobacillus* database^6^ (Niderman-Meyer et al., 2010; Deng et al., 2013). The identification of starch-binding proteins by LC-ESI-MS/MS was completed at Beijing Protein Innovation, Co., Ltd (Beijing, China).

### Statistical Analysis

Data were obtained in triplicate and are reported as averages; Statistical analyses were performed to determine significant differences (*p* < 0.05) among obtained results using the Student’s *t*-test or ANOVA followed by Duncan’s multiple range test. All data were analyzed using the SPSS 16 software (SPSS, Chicago, IL, United States).

## Results

### Microbial Counts in the Sweet Potato Sour Liquid

The counts of bacteria was the highest (8.96 ± 0.01 log CFU mL^-1^). Yeasts were 5.04 ± 0.04 log CFU mL^-1^. Furthermore, molds were 2.71 ± 0.02 log CFU mL^-1^ and the lowest number in sour liquid. Bacteria, therefore, were dominant in the sour liquid.

### Bacterial Composition in the Sour Liquid Determined by 16S rDNA Sequencing

**FIGURE 1:**
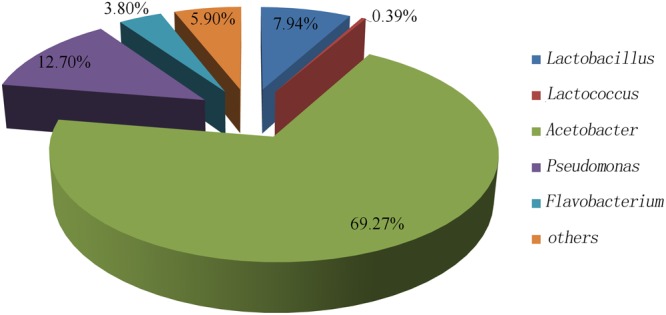
The sour liquid bacterial community at genus level.

To investigate the dominant bacteria in sour liquid, bacterial composition in the sour liquid was evaluated using high-throughput sequencing of the V4 regions of 16S rRNA genes. The bacterial community was analyzed at the genus level by comparing with Greengenes reference database. *Acetobacter* species were dominant in the liquid, accounting for 69.27% of bacteria in sour liquid; *Pseudomonas* species accounted for 12.70% of bacteria in sour liquid; while *Lactobacillus* and *Lactococcus* species accounted for only 7.94 and 0.39% of bacteria in sour liquid, respectively (**Figure 1**). We conclude that *Acetobacter, Pseudomonas*, and *Lactobacillus* are the dominant bacteria in the sour liquid.

### Isolation of Flocculating Strains

In total, 86 bacteria, 20 yeasts, and 10 molds were isolated from the sour liquid on TJA and Rose-Bengal media; they were inoculated and cultured in the sweet potato juice medium to identify strains with flocculating activity. Eight strains exhibited flocculating activity. These eight isolates were all bacterial strains, with a rod shape and chain-like arrangement of cells, and were identified as *Lactobacillus* by 16S rDNA sequence homology comparisons. The yeasts and molds did not show any starch-flocculating activity, indicating that *Lactobacillus* was the dominant flocculating microorganism in the sweet potato sour liquid. The most pronounced flocculating activity among the flocculating strains was observed during the fermentation with *Lactobacillus* strain L1. Strain L1 was subsequently identified as *L. paracasei* subsp. *paracasei* based on its colony character, morphological, and physiological characteristics, as well as 16S rDNA sequence homology referencing Bergey’s Manual of Systematic Bacteriology; accordingly, it was named *L. paracasei* subsp. *paracasei* L1. This strain was deposited in the China General Microbiological Culture Collection Center (CGMCC, no. 4163).

### Distribution of the Flocculating Activity in Cell Culture

**FIGURE 2:**
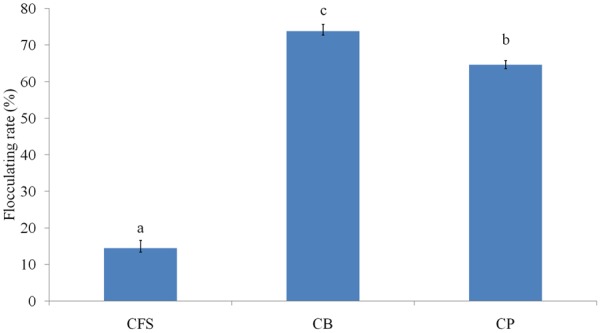
Distribution of the flocculating activity in *Lactobacillus paracasei* subsp. *paracasei* L1 cultures. CB, culture broth; CFS, cell-free supernatant; CP, cell pellet (cells harvested from the culture and resuspended in PBS). Columns with different letters indicate statistical significance (*p* < 0.05).

The distribution of flocculating activity in cell culture, i.e., its association with the cells and extracellular secretions of *L. paracasei* subsp. *paracasei* L1, was investigated. It was conclude that more than 85% of the flocculating activity was cell-associated, and less than 15% of the activity was associated with the extracellular secretions (**Figure 2**).

### Changes of Starch Granule Size in Suspension Associated with a Treatment with *L. paracasei* subsp. *paracasei* L1 Cultures

**FIGURE 3:**
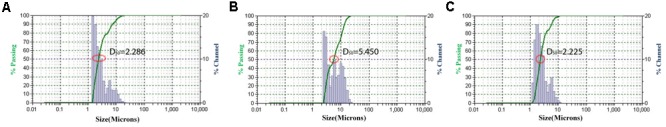
The effect of flocculation on starch granule size. **(A)** Particle size of starch granules in starch suspension before flocculation. **(B)** Particle size of starch granules in starch deposition after flocculation. **(C)** Particle size of starch granules in the supernatant after flocculation.

The size of starch granules before and after treatment with *L. paracasei* subsp. *paracasei* L1 cultures was evaluated with Microtrac S3500 laser grain size analyzer. The average particle size, D_50_, also called the median diameter, denotes a cumulative 50% point of diameter (or 50% pass particle size). The D_50_ (the average particle size) of starch granule size increased, from 2.286 to 5.450 μm, in the presence of *L. paracasei* subsp. *paracasei* L1 cells, suggesting that the starch granules formed massive floccules (**Figure 3**).

### Microscopic Observation of Starch Granules Flocculated by *L. paracasei* subsp. *paracasei* L1 Cultures

**FIGURE 4:**
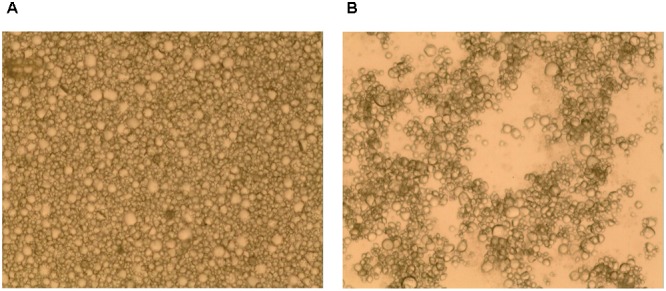
Optical micrograph of starch granule aggregation. Sweet potato starch milk before **(A)** and after **(B)** the addition of *L. paracasei* subsp. *paracasei* L1 cultures (100×).

**FIGURE 5:**
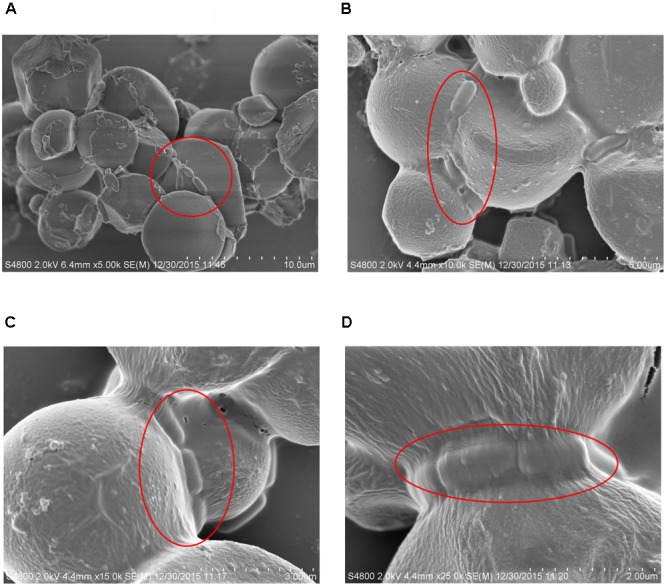
Scanning electron micrograph (SEM) of starch granules with the adhering *L. paracasei* subsp. *paracasei* L1 cells. **(A)** 5,000×; **(B)** 1,000×; **(C)** 15,000×; **(D)** 25,000×.

The size distribution of starch granules before the addition of bacteria was homogeneous and uniform, as observed under an optical microscope at a magnification of 100× (**Figures 4A,B**). In the presence of bacteria, the starch granules rapidly aggregated and formed massive floccules. Furthermore, SEM analysis revealed that the *L. paracasei* subsp. *paracasei* L1 cells adhered to the surface of starch granules (**Figures 5A–D**). Multiple bacterial cells adhered to starch granules surface and also to each other, thus forming bridge-like structures linking starch granules and forming the aggregated floc.

### ζ Potential of Solutions during Flocculation

ζ Potential of the starch suspension was initially a lot lower than that of *L. paracasei* subsp. *paracasei L1* cell suspension in sweet potato juice medium (**Table 1**). After the addition of cells to the starch suspension (10%, v/v), the potential was closer to zero. According to the DLVO theory, this indicated that the starch granules were in a very unstable state and readily formed a floc precipitation. Both the starch and the cells were negatively charged in water, indicating that the starch-bacterium adhesion was not effected by electrostatic interactions.

**Table 1:** Changes in the ζ potential during flocculation.

Sample	ζ Potential (mV)
Starch suspension	–13.97 ± 0.23^a^
Cultures of L1	–0.49 ± 0.02^c^
Starch suspension with adding 10% cultures of L1	–2.29 ± 0.02^b^

*Values are presented as means ± SD. Values with different letter designations within the same row are significantly different (*p* < 0.05).*

### The Effect of Physical, Chemical, and Enzymatic Treatments of *L. paracasei* subsp. *paracasei* L1 Cells on Their Flocculating Ability

**FIGURE 6:**
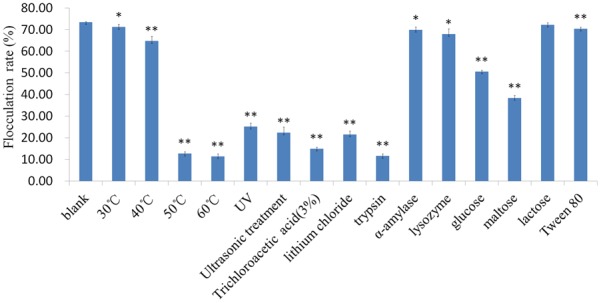
Effects of various treatments of L1 cells on their flocculation. Differences between FR with cells after various treatments and control group were analyzed statistically using *t*-test. ^∗^*p* < 0.05, ^∗∗^*p* < 0.01.

The effect of various treatments on the flocculating ability of L1 cells was investigated to determine the nature of the flocculation factor on the cell surface, and the nature of the interacting force between the cells and starch granules. As shown in **Figure 6**, the flocculating activity was sensitive to heat treatment, but not to α-amylase or lysozyme treatments, indicating that the surface polysaccharide of *L. paracasei* subsp. *paracasei* L1 did not mediate the flocculation. In contrast, the flocculating activity of cells was affected by UV, ultrasonic treatment, trichloroacetic acid, and lithium chloride, which are all protein denaturants. Furthermore, trypsin treatment significantly reduced the flocculating activity of cells. Collectively, these results indicated that bacterial surface proteins play a role in flocculation.

Flocculation was slightly affected by Tween 80. This suggested an absence of hydrophobic interactions between *L. paracasei* subsp. *paracasei* L1 cells and starch granules; similarly, electrostatic interactions did not occur because both starch granules and cells were negatively charged in water (*vide supra*). Moreover, these interactions were likely specific because glucose and maltose significantly inhibited the flocculation (**Figure 6**).

### Identification of Candidate Starch-Binding Proteins

Surface proteins from *L. paracasei* subsp. *paracasei* L1 extracted by the guanidine hydrochloride method were incubated with starch granules. After a series of non-specific washes, proteins adhering to the starch granules were removed by re-suspension in PBS containing 100 mM maltose. Ten candidate starch-binding proteins were then identified by LC-ESI-MS/MS: three of these proteins were glycolytic enzymes; two were identified as the translation elongation factor Tu; one was a cell wall hydrolase; one was a surface antigen; one was lyzozyme M1; one was a glycoside hydrolase; and one was an uncharacterized proteins (**Table 2**). Mascot score for these hits was >100. None of the proteins were detected in the control.

**Table 2:** Candidate starch-binding proteins.

Protein	Accession number	MM/pI	Seq cov/pep match	Mascot score
Cell wall hydrolase	tr| S2N653	41513/8.93	25/11	455
Surface antigen	tr| A0A0C9P9Z1	42463/6.97	9/6	199
Phosphoglycerate kinase	tr| K6QCV8	39603/5.51	16/11	311
Enolase	tr| A0A0C9Q4L1	47058/4.73	9/8	234
Elongation factor Tu	tr| A0A0C9PFS8	43546/4.87	12/11	232
Elongation factor Tu	sp| Q88VE0	43350/4.95	7/7	131
Lyzozyme M1 (1,4-beta-*N*-acetylmuramidase)	tr| S2NSV8	100512/6.74	9/7	183
Glyceraldehyde-3-phosphate dehydrogenase	tr| A0A0C9PWL0	36912/5.68	3/3	139
Uncharacterized protein	tr| A0A0F4KSC0	41048/8.59	11/10	130
Glycoside hydrolase	tr| A0A0B8U0A4	49407/4.93	7/4	105

## Discussion

Sour liquid, whether from sweet potato or mung bean, is used as a microbial flocculant and plays a role in promoting the precipitation of starch during preparation for starch (Research Groups of Sour Liquid, 1974; Xu and Liu, 1980). Nevertheless, data on the dominant flocculating microbes are inconsistent. *Acetobacter, Lactobacillus*, and *Pseudomonas* are dominant in the sweet potato sour liquid, yet all the strains screened in the current study that exhibited flocculating activity belonged to the *Lactobacillus* genus. Notably, *Lactobacillus* sp. were also the first species discovered to flocculate starch (Research Groups of Sour Liquid, 1974; Wei and Qun, 2007). On the other hand, *L. lactis* is responsible for starch flocculation and no other microorganisms has the ability to flocculate starch in mung bean sour liquid (Research Groups of Sour Liquid, 1974; Xu and Liu, 1980; Wei and Qun, 2007). *Lactococcus* is also present in the sweet potato sour liquid but was not isolated in the current study, perhaps because of its low numbers. The difference in nutritional components of the sweet potato and mung bean may account for the discrepancy in dominant bacteria responsible for the flocculation. *L. paracasei* subsp. *paracasei* L1 and *L. lactis* have some common features, which may be associated with their flocculating activity. First, both are lactic acid bacteria that can decrease the pH of the sour liquid. The acidic environment is considered indispensable for facilitation of the flocculation of starch in sour liquid. Normally, the sour liquid flocculating activity peaks at pH 4.5 (Research Groups of Sour Liquid, 1974). Second, the cells of both species were arranged in a chain after cell division. The flocculating activity is high when the microbial flocculant forms linear higher-order structures. In contrast, the flocculating activity is low when the microbial flocculant has a branching structure (Biggs et al., 2000; Brostow et al., 2007; Hjorth and Jørgensen, 2012). In the current study, the chain-like arrangement of bacterial cells facilitated starch flocculation.

According to the distribution of flocculating activity, the microbial flocculant may be generally classified into two groups: one located on the microbial cell surface, and one in the culture solution (Brostow et al., 2007; Bhattacharya et al., 2017; Liu et al., 2017). In the current study, more than 85% of the flocculating activity was associated with the L1 cells, while less than 15% of the activity was associated with the culture liquid. The L1 cells and starch granules interacted via specific, rather than electrostatic or hydrophobic, interactions because both the cells and granules are negatively charged in water. Furthermore, the flocculating activity of L1 cells was visibly inhibited by glucose or maltose, but was not precise by Tween 80. As such, the ζ potential and repulsion decreased from -13.97 ± 0.23^a^ mv to -2.29 ± 0.02^b^ mv. Multiple L1 cells adhere to a single starch granule. Subsequently, many starch granules were connected by L1 cells that served as bridging agents coagulating the starch granules, thereby increasing the starch granule size, and resulting in the formation of massive flocs and easy deposition. During flocculation, the chain-like arrangement contributed to starch precipitation. Therefore, the flocculation of starch by these cells was consistent with the bridging mechanism that is essential for microbial flocculants shown in other studies (Aljuboori et al., 2016; Raj et al., 2016; Du et al., 2017).

It is prerequisite for the flocculation that *L. paracasei* subsp. *paracasei* L1 adhere to starch. Other bacteria, such as *Bifidobacterium* species, *V. cholerae, B. thetaiotaomicron*, and *L. amylovorus*, also adhere to starch granules; the mechanisms of their adhesion all appear to involve cell surface proteins (Reeves et al., 1996; Crittenden et al., 2001; Ryan et al., 2006; Niderman-Meyer et al., 2010). In the current study, based on SEM observations, the adhesion factors were located on L1 cell surface. The chemical component of the flocculating factors was then evaluated by physical, chemical, and enzymatic treatments of cells, to verify whether cell surface proteins rather than whole cell peptidoglycan or other polysaccharides of *L. paracasei* subsp. *paracasei* L1 were involved in the adhesion.

We identified 10 candidate proteins that were involved in the L1 cell-starch interaction; most of them were known to function as adhesins on the cell surface of intestinal bacteria. The identified cell wall hydrolase had the highest Mascot score (455), which indicated that this protein was highly likely to be as identified. Cell wall hydrolases catalyze the cleavage of peptidoglycan sugar or peptide chains (Claes, 2012). Similarly, lysozyme M1 (1,4-β-*N*-acetylmuramidase) was detected on the L1 cell surface (Mascot score of 183). These hydrolases play important roles in the regulation of cell wall growth, turnover, and maintenance, and in the separation of daughter cells. Hydrolase is also found on the cell wall of *Lactobacillus rhamnosus* GG (LGG), often near the mature septa of exponential cells, exhibiting D-glutamyl-L-lysyl endopeptidase activity in zymogram assays (Nadkarni et al., 2014). Mutation of the cell wall hydrolase in LGG impedes normal separation of daughter cells and the cells are arranged in rather long and overly extended chains (Claes, 2012; Smokvina et al., 2013; Nadkarni et al., 2014). In this bacterium, the hydrolysis of muropeptides in the cell wall also likely affects daughter cell separation and regulates the length of the cell chain structure. The unusual chain structure might reflect the increase in steric hindrance that effectively blocks the interaction between the bacterial surface and starch granules. Hence, appropriate chain length regulated by cell wall hydrolase might aid the flocculation of starch by the bridging mechanism. In the current study, we show for the first time the involvement of this protein in the adhesion of *L. paracasei* subsp. *paracasei* to starch granules.

Four identified starch-binding proteins were associated with glucose metabolism. Three of them were glycolytic enzymes, namely, phosphoglycerate kinase (PGK, Mascot score 311), enolase (ENO, 234), and glyceraldehyde-3-phosphate dehydrogenase (GAPDH, 139); the fourth one was identified as a glycoside hydrolase (score 139). These sugar-metabolizing enzymes are found in most bacterial cells and play a role in sugar catabolism or degradation of such complex carbohydrates as lactose or starch (Ramiah et al., 2008; Glenting et al., 2013). We asked how these glycolytic enzymes and glycoside hydrolase promote the adhesion of L1 cells to starch granules. On the one hand, the starch granules act as stable surfaces in starch milk, and might facilitate the adhesion of L1 cells because bacteria prefer to grow on solid surfaces rather than in the surrounding aqueous phase (Zobell, 1943; Bäckhed et al., 2005; Boone and Tyrrell, 2012). On the other hand, these proteins, as sugar-metabolizing enzymes, might be available to degrade starch or the products of its decomposition if they are also involved in cellular adhesion, providing energy and sustaining bacterial survival. Corn starch that is flocculated by the sour liquid has low amylase content and small-volume average granule size, high swelling capacity, and high solubility, which suggests that starch is metabolized by bacteria in the sour liquid (Chang et al., 2006; Li et al., 2008). Consequently, the presence of glycolytic enzymes and glycoside hydrolase on L1 cell surface could play a role in acquiring and metabolizing starch. SEM analyses of the intestines of mice maintained on a standard high-polysaccharide chow diet revealed that the bacterial communities assemble on small undigested or partially digested food particles (Bäuerl et al., 2010). Glycolytic enzymes and glycoside hydrolase are produced by *B. thetaiotaomicron*, a prominent mutualist in the distal intestine of adult human (Lebeer, 2010). Whole-genome transcriptional profiling of *B. thetaiotaomicron* revealed that a high-polysaccharide chow diet is associated with a selective up-regulation of a subset of SusC and SusD paralogs that bind to and import starch, a subset of glycoside hydrolases, and genes encoding enzymes involved in the delivery of mannose, galactose, and xylose to the pentose phosphate pathway (Liu and Shen, 2007a,b; Deng et al., 2013; Li et al., 2015). Similarly, adhesion to starch might facilitate the hydrolysis of starch and its products, including glucose, by glycolytic enzymes and glycoside hydrolase located either on the cell surface or inside L1 cells. Hence, the bacterium may efficiently use starch and colonize it, surviving under these conditions.

## Conclusion

As determined by 16S rDNA sequencing and traditional microbiology techniques, *Lactobacillus* was the dominant flocculating bacterial genus in the sweet potato sour liquid. *L. paracasei* subsp. *paracasei* L1 strain with a high flocculating activity was isolated, and the flocculation mechanism of its adhesion to starch was investigated. Our results showed that the *L. paracasei* subsp. *paracasei* L1 cells specifically bound starch granules and linked these starch granules to form large flocs by bridging. This accelerated starch deposition. The starch-binding proteins on the surface of *L. paracasei* subsp. *paracasei* L1 cells were extracted using guanidine hydrochloride, and 10 proteins with Mascot scores ≥ 100 were identified by mass spectrometry. These proteins are also present, as adhesion molecules, on the cell surface of other probiotic bacteria. Their role in bacterial starch metabolism, functional properties, and potential applications in adhesion to starch or other materials should be further investigated.

## Availability of Data and Material

Eight strains exhibited flocculating activity. These eight isolates were all bacterial strains, and were identified as *Lactobacillus* by 16S rDNA sequence homology comparisons. Sequences of this project have been deposited in the NCBI sequence read archive (https://www.ncbi.nlm.nih.gov/genbank/) under GenBank accession numbers: KY952217 (L1); KY952218 (LL1); KY978461 (L28); KY978462 (L36); KY978463 (L53); KY978464 (S07); KY978465 (S10); KY978466 (S32).

## Author Contributions

LZ, YX, and XhL performed all experiments and wrote the paper. YY, XoL, HZ, and ZZ conducted the experiments and data analysis. All authors read and approved the manuscript.

## Conflict of Interest Statement

The authors declare that the research was conducted in the absence of any commercial or financial relationships that could be construed as a potential conflict of interest. The reviewer GP and handling Editor declared their shared affiliation, and the handling Editor states that the process nevertheless met the standards of a fair and objective review.

## Footnotes

1 http://ccb.jhu.edu/software/FLASH/

2 http://www.mothur.org/

3 http://qiime.org/scripts/pick_otus.html

4 http://greengenes.secondgenome.com/

5 www.ncbi.nlm.nih.gov/blast

6 http://www.uniprot.org/taxonomy/1578

## References

[B1] Aljuboori A. H. R. Uemura Y. Thanh N. T. (2016). Flocculation and mechanism of self-flocculating lipid producer microalga *Scenedesmus quadricauda*, for biomass harvesting. *Biomass Bioenergy* 93 38–42. 10.1016/j.biombioe.2016.06.013

[B2] Anastasi A. Varese G. C. Marchisio V. F. (2005). Isolation and identification of fungal communities in compost and vermicompost. *Mycologia* 97 33–44. 10.1080/15572536.2006.11832836 16389954

[B3] Bäckhed F. Ley R. E. Sonnenburg J. L. Peterson D. A. Gordon J. I. (2005). Host-bacterial mutualism in the human intestine. *Science* 307 1915–1920. 10.1126/science.1104816 15790844

[B4] Bäuerl C. Pérezmartínez G. Yan F. Polk D. B. Monedero V. (2010). Functional analysis of the p40 and p75 proteins from *Lactobacillus casei* bl23. *J. Mol. Microbiol. Biotechnol.* 19 231–241. 10.1159/000322233 21178363 PMC3019367

[B5] Beck H. C. Madsen S. M. Glenting J. Petersen J. Israelsen H. Nørrelykke M. R. (2009). Proteomic analysis of cell surface-associated proteins from probiotic *Lactobacillus plantarum*. *FEMS Microbiol. Lett.* 297 61–66. 10.1111/j.1574-6968.2009.01662.x 19527296

[B6] Bhattacharya A. Mathur M. Kumar P. Prajapati S. K. Malik A. (2017). A rapid method for fungal assisted algal flocculation: critical parameters & mechanism insights. *Algal Res.* 21 42–51. 10.1016/j.algal.2016.10.022

[B7] Biggs S. Habgood M. Jameson G. J. Yan Y. D. (2000). Aggregate structures formed via a bridging flocculation mechanism. *Chem. Eng. J.* 80 13–22. 10.1016/S1383-5866(00)00072-1

[B8] Blanton L. V. Charbonneau M. R. Salih T. Barratt M. J. Venkatesh S. Ilkaveya O. (2016). Gut bacteria that prevent growth impairments transmitted by microbiota from malnourished children. *Science* 351:aad3311 10.1126/science.aad3311 PMC4787260 26912898

[B9] Boone T. J. Tyrrell G. J. (2012). Identification of the actin and plasminogen binding regions of group b streptococcal phosphoglycerate kinase. *J. Biol. Chem.* 287 29035–29044. 10.1074/jbc.M112.361261 22761440 PMC3436549

[B10] Brostow W. Pal S. Singh R. P. (2007). A model of flocculation. *Mater. Lett.* 61 4381–4384. 10.1016/S1383-5866(00)00072-1

[B11] Chang Y. H. Lin C. L. Chen J. C. (2006). Characteristics of mung bean starch isolated by using lactic acid fermentation solution as the steeping liquor. *Food Chem.* 99 794–802. 10.1016/j.foodchem.2005.07.060

[B12] Claes I. J. (2012). Genetic and biochemical characterization of the cell wall hydrolase activity of the major secreted protein of *Lactobacillus rhamnosus* GG. *PLoS ONE* 7:e31588 10.1371/journal.pone.0031588 PMC3281093 22359601

[B13] Coombs J. T. Franco C. M. M. (2003). Isolation and identification of actinobacteria from surface-sterilized wheat roots. *Appl. Environ. Microbiol.* 69 5603–5608. 10.1128/AEM.69.9.5603-5608.2003 12957950 PMC194995

[B14] Crittenden R. Laitila A. Forssell P. Mättö J. Saarela M. Mattila-Sandholm T. (2001). Adhesion of bifidobacteria to granular starch and, its implications in probiotic technologies. *Appl. Environ. Microbiol.* 67 3469–3475. 10.1128/AEM.67.8.3469-3475.2001 11472921 PMC93045

[B15] Deng F. M. Mu T. H. Zhang M. Abegunde O. K. (2013). Composition, structure, and physicochemical properties of sweet potato starches isolated by sour liquid processing and centrifugation. *Starch* 65 162–171. 10.1002/star.201200106

[B16] Donaldson G. P. Lee S. M. Mazmanian S. K. (2016). Gut biogeography of the bacterial microbiota. *Nat. Rev. Microbiol.* 14 20–32. 10.1038/nrmicro3552 26499895 PMC4837114

[B17] Dong H. C. An S. M. Chun S. Yang E. C. Selph K. E. Lee C. M. (2015). Dynamic changes in the composition of photosynthetic picoeukaryotes in the northwestern Pacific Ocean revealed by high-throughput tag-sequencing of plastid 16s rRNA genes. *FEMS Microbiol. Ecol.* 17:fiv170 10.1093/femsec/fiv170 26712350

[B18] Du Q. Wei H. Li A. Yang H. (2017). Evaluation of the starch-based flocculants on flocculation of hairwork wastewater. *Sci. Total Environ.* 60 1628–1637. 10.1016/j.scitotenv.2017.06.029 28609850

[B19] Glenting J. Beck H. C. Vrang A. Riemann H. Ravn P. Hansen A. M. (2013). Anchorless surface associated glycolytic enzymes from *Lactobacillus plantarum*, 299v bind to epithelial cells and extracellular matrix proteins. *Microbiol. Res.* 168 245–253. 10.1016/j.micres.2013.01.003 23395591

[B20] Hjorth M. Jørgensen B. U. (2012). Polymer flocculation mechanism in animal slurry established by charge neutralization. *Water Res.* 46 1045–1051. 10.1016/j.watres.2011.11.078 22196952

[B21] Lebeer S. (2010). Host interactions of probiotic bacterial surface molecules: comparison with commensals and pathogens. *Nat. Rev. Microbiol.* 8 171–184. 10.1038/nrmicro2297 20157338

[B22] Li X. Wang C. Lu F. Zhang L. Yang Q. (2015). Physicochemical properties of corn starch isolated by acid liquid and l -cysteine. *Food Hydrocoll.* 44 353–359.

[B23] Li Z. Liu W. Shen Q. Wei Z. Tan B. (2008). Properties and qualities of vermicelli made from sour liquid processing and centrifugation starch. *J. Food Eng.* 86 162–166. 10.1016/j.jfoodeng.2007.09.013

[B24] Lian B. Chen Y. Zhao J. Teng H. H. Zhu L. Yuan S. (2008). Microbial flocculation by bacillus mucilaginosus: applications and mechanisms. *Bioresour. Technol.* 99 4825–4831. 10.1016/j.biortech.2007.09.045 17967531

[B25] Lin W. H. Hwang C. F. Chen L. W. Tsen H. Y. (2006). Viable counts, characteristic evaluation for commercial lactic acid bacteria products. *Food Microbiol.* 23 74–81. 10.1016/j.fm.2005.01.013 16942989

[B26] Liu W. Shen Q. (2007a). Structure analysis of mung bean starch from sour liquid processing and centrifugation. *J. Food Eng.* 79 1310–1314. 10.1016/j.jfoodeng.2006.04.012

[B27] Liu W. Shen Q. (2007b). Studies on the physicochemical properties of mung bean starch from sour liquid processing and centrifugation. *J. Food Eng.* 79 358–363. 10.1016/j.jfoodeng.2006.01.065

[B28] Liu Z. Wei H. Li A. Yang H. (2017). Evaluation of structural effects on the flocculation performance of a co-graft starch-based flocculant. *Water Res.* 118 160–166. 10.1016/j.watres.2017.04.032 28431348

[B29] Muyanja C. M. Narvhus J. A. Treimo J. Langsrud T. (2003). Isolation, characterisation and identification of lactic acid bacteria from bushera: a Ugandan traditional fermented beverage. *Int. J. Food Microbiol.* 80 201–210. 10.1016/S0168-1605(02)00148-4 12494920

[B30] Nadkarni M. A. Chen Z. Wilkins M. R. Hunter N. (2014). Comparative genome analysis of *Lactobacillus rhamnosus* clinical isolates from initial stages of dental pulp infection: identification of a new exopolysaccharide cluster. *PLoS ONE* 9:e90643 10.1371/journal.pone.0090643 PMC3954586 24632842

[B31] Niderman-Meyer O. Zeidman T. Shimoni E. Kashi Y. (2010). Mechanisms involved in governing adherence of *Vibrio cholerae* to granular starch. *Appl. Environ. Microbiol.* 76 1034–1043. 10.1128/AEM.01533-09 20023099 PMC2820958

[B32] O’Riordan K. Muljadi N. Conway P. (2001). Characterization of factors affecting attachment of bifidobacterium species to amylomaize starch granules. *J. Appl. Microbiol.* 90 749–754. 10.1046/j.1365-2672.2001.01304.x 11348435

[B33] O’Toole G. A. (2016). Classic spotlight: *Bacteroides thetaiotaomicron*, starch utilization, and the birth of the microbiome era. *J. Bacteriol.* 198 2763 10.1128/JB.00615-16 PMC5037999 27660335

[B34] Piotrowska A. Gosiewski T. Bulanda M. Brzychczy-Wloch M. (2016). Using of the 16S rDNA sequencing for identification of *Lactobacillus* species. *Med. Dosw. Mikrobiol.* 2016 5–11. 28146617

[B35] Raj P. Batchelor W. Blanco A. de la Fuente E. Negro C. Garnier G. (2016). Effect of polyelectrolyte morphology and adsorption on the mechanism of nanocellulose flocculation. *J. Colloid Interface Sci.* 481 158–167. 10.1016/j.jcis.2016.07.048 27474816

[B36] Ramiah K. van Reenen C. A. Dicks L. M. (2008). Surface-bound proteins of *Lactobacillus plantarum* 423 that contribute to adhesion of caco-2 cells and their role in competitive exclusion and displacement of *Clostridium sporogenes* and *Enterococcus faecalis*. *Res. Microbiol.* 159 470–475. 10.1016/j.resmic.2008.06.002 18619532

[B37] Reeves A. R. D’Elia J. N. Frias J. Salyers A. A. (1996). A *Bacteroides thetaiotaomicron* outer membrane protein that is essential for utilization of maltooligosaccharides and starch. *J. Bacteriol.* 178 823–830. 10.1128/jb.178.3.823-830.1996 8550519 PMC177731

[B38] Reeves A. R. Wang G. R. Salyers A. A. (1997). Characterization of four outer membrane proteins that play a role in utilization of starch by *Bacteroides thetaiotaomicron*. *J. Bacteriol.* 179 643–649. 10.1128/jb.179.3.643-649.1997 9006015 PMC178742

[B39] Research Groups of Sour Liquid (1974). Why can sour liquid precipitate starch? *Acta Sci. Nat. Univ. Pekinensis* S1 57–66. 10.13209/j.0479-8023.1974.040

[B40] Rodriguez-Sanoja R. Oviedo N. Sanchez S. (2005). Microbial starch-binding domain. *Curr. Opin. Microbiol.* 8 260–267. 10.1016/j.mib.2005.04.013 15939348

[B41] Ryan S. M. Fitzgerald G. F. Sinderen D. V. (2006). Screening for and identification of starch-, amylopectin-, and pullulan-degrading activities in bifidobacterial strains. *Appl. Environ. Microbiol.* 72 5289–5296. 10.1128/AEM.00257-06 16885278 PMC1538741

[B42] Shipman J. A. Berleman J. E. Salyers A. A. (2000). Characterization of four outer membrane proteins involved in binding starch to the cell surface of *Bacteroides thetaiotaomicron*. *J. Bacteriol.* 182 5365–5372. 10.1128/JB.182.19.5365-5372.2000 10986238 PMC110978

[B43] Smokvina T. Wels M. Polka J. Chervaux C. Brisse S. Boekhorst J. (2013). *Lactobacillus paracasei* comparative genomics: towards species pan-genome definition and exploitation of diversity. *PLoS ONE* 8:e68731 10.1371/journal.pone.0068731 PMC3716772 23894338

[B44] Wei Z. Qun S. (2007). The effects of chemical pre-treatment of one precipitating bacteria and *Lactococcus lactis* As1.9 on their starch precipitating ability. *Sci. Technol. Food Ind.* 28 123–126.

[B45] Xu H. Liu M. L. (1980). A starch-agglutinating factor on the cell wall of *Streptococcus lactis* strain? Electron microscopic observation. *Acta Microbiol. Sin.* 20 276–279. 10.13343/j.cnki.wsxb.1980.03.009

[B46] Xu Y. Yang H. Zhang L. Su Y. Shi D. Xiao H. (2016). High-throughput sequencing technology to reveal the composition and function of cecal microbiota in Dagu chicken. *BMC Microbiol.* 16:259 10.1186/s12866-016-0877-2 PMC5097418 27814685

[B47] Zobell C. E. (1943). The effect of solid surfaces upon bacterial activity. *J. Bacteriol.* 46 39–56. 16560677 10.1128/jb.46.1.39-56.1943 PMC373789

